# Editorial: From “modern” to “postmodern” psychology: Is there a way past?

**DOI:** 10.3389/fpsyg.2023.1091721

**Published:** 2023-03-03

**Authors:** Barbara Hanfstingl, Jana Uher, Peter A. Edelsbrunner, Ulrich Dettweiler, Timo Gnambs

**Affiliations:** ^1^Institute for School and Instructional Development, University of Klagenfurt, Klagenfurt, Austria; ^2^School of Human Sciences, University of Greenwich, London, United Kingdom; ^3^Department of Humanities, Political and Social Sciences, ETH Zürich, Zürich, Switzerland; ^4^Faculty of Arts and Education, University of Stavanger, Stavanger, Norway; ^5^Educational Measurement, Leibniz Institute for Educational Trajectories (LIfBi), Bamberg, Germany

**Keywords:** modern psychology, postmodern psychology, methodology, psychology of science, epistemology in psychology, psychological phenomena and processes

Contemporary psychology is facing profound problems and various obstacles to advancing its research, as reflected in its continued crises in replicability, confidence, generalizability, and validity. “Modern” paradigms–involving beliefs in determinative cause-effect relations between the elements of an objectively given world, which are thus amenable to experimental, rational exploration and mostly linear statistical analyses–often no longer do justice to the complexity of psychology's contemporary research questions. Critical analyses of established concepts and approaches have not yet been sufficiently considered in mainstream theorizing nor have adequate consequences been drawn from them to advance our understanding of the phenomena of mind and behavior as well as to elaborate overarching frameworks and to further methodologies and methods that are suited for their exploration.

This Research Topic assembled contributions from authors with expertise in different specialties to work toward developing a new understanding of psychological science, aimed at tackling current problems and devising possible solutions by exploring the promises of “postmodernism” as well as of further epistemologies and research paradigms beyond. In the context of science, “postmodernism” has no overarching meaning. It is associated with epistemological developments after Karl Popper's critical rationalism, such as constructivism, systemic approaches, and epistemological as well as a methodological plurality. To avoid fruitless doctrinal dispute, we did not insist on the terms “modern” and “postmodern” nor on any narrow definition of them. Instead, we invited papers proposing new ideas and solutions that may have the potential to tackle the epistemological, conceptual, and methodological challenges of “modern” psychology and to improve research quality through more critical and more in-depth reconsiderations than commonly done in currently popular calls for “robust analysis,” preregistration, replicability, and open science. Our key questions were to what extent we need to abandon the ideas of critical rationalism, to what extent we need to integrate concepts and methodological strategies from other disciplines, and to what extent we should focus on entirely new problem-solving strategies.

The current Research Topic includes 15 articles from different world regions that have discussed these issues and key questions from multiple perspectives. Here, we briefly summarize these contributions to highlight their diversity as well as central themes in their future-oriented reflections and proposals for solutions.

Holtz contrasts postmodernist with critical rationalist conceptualizations and analyses the main differences. He shows that, rather unexpectedly, Karl Popper's and David Deutsch's understanding of objective knowledge, progress, and methods is not that different from Kenneth Gergen's understanding. All of them agree that focusing only on a specific scientific method neither justifies nor validates psychological knowledge. Popper and Deutsch see scientific progress in the formulation of “better” theories, which are derived from formal and logical reasoning, whereas Gergen sees scientific progress much more in its abilities to address real-world problems in the context of culture and society. Holtz argues for a joined next step for developing epistemology in psychological science.

In a similar vein, Mazur revives early criticisms of positivism that has been voiced in pre-postmodernist times already. He suggests that, rather than addressing the shortcomings of positivistic epistemology by means of postmodernism, psychology would be better served by the deeper, more consequential reflections of Sapientia, a form of metaphysical wisdom that asserts the power of science as a method, while also critically and cautiously supporting the polyvalence and complexities of life as highlighted in postmodern thought.

Veraksa et al. propose dialectical thinking as a basis for developing psychology from a modern to a postmodern science. Dialectical thinking recognizes the importance of contradiction, change and synthesis. This includes recognizing the values as well as the limitations of modern epistemological approaches, such as both universalistic formal analysis (often associated with modernist approaches) and relativistic analysis (often associated with the rejection of modernist approaches). Veraksa et al. present dialectic thinking as a powerful processual approach to conceiving scientific thinking and advancing the development of scientific knowledge.

Iso-Ahola points out that the scientific truth that we aspire has to be seen generally as time-related, context-related, and method-related. A successful replication does not automatically mean validation of findings when simple measurement problems, like the reliability of a scale, remain unconsidered. He further points to the influence of methodical artifacts, stability, temporality, context-dependence, and the implicity of many psychological phenomena, which all disturb the accuracy of psychological constructs. Therefore, Iso-Ahola suggests focusing on psychological phenomena in replication studies and evaluating them primarily on a theoretical level rather than only on a methodical level.

Krueger highlights the mutual enrichment that is needed between forward-looking experimental psychology and backward-looking historical psychology in a postmodern scientific era, given that prediction and explanation make no sense without the other. In other words, they can be seen as the same, only the direction of the time flow is reversed. Krueger supports his argument with Bayesian considerations and the diagnostic ratio, showing that probability and effectiveness are inversely related so that rare causes with high effectiveness must also be considered in psychological explanatory models. He supports this argument with thought experiments based on the three historical case studies of Philipp von Hutten, Gonzalo Guerrero, and Robinson Crusoe.

Another fundamental statement comes from Rabeyron. He uncovers problems of psychological mainstream methodology with particular contents in psychology that are associated with findings that have led to the Nobel Prize in physics 2022 for research in quantum entanglement by Alain Aspect, John Clauser, and Anton Zeilinger. Based on two examples of psi research, the Ganzfeld experiment and the Bem experiment, Rabeyron argues that much developmental work on methodology and theory has to be done to explain psychological phenomena that are as complex as psi is assumed to be. Rabeyron connects his considerations with earlier work that is explicitly associated with physics, for example, Lucadou's Model of Pragmatic Information.

Focusing on knowledge generation in psychological experiments, Mayrhofer et al. analyze their underlying philosophy of science. They state that researchers must reduce and pre-structure the phenomena of interest in order to (re)create narrowly defined phenomena in a controlled environment and develop meaningful research questions and hypotheses. That is, rather than a copy of “reality,” the experimental setup is an active construction by the researchers and reflects their pre-experimental understanding. Mayrhofer et al. demonstrate that postmodern concepts have always been at the heart of psychological experiments and can therefore be fruitfully applied to sharpen the theoretical and empirical basis of experiments.

From a more societal perspective, Guyon highlights the tension that exists between the scientific imperative of quantification in experimental psychology and the social imperative of its actual use and implementation in psychology. Specifically, standardization, control and regulation are meant to provide scientifically validated findings that serve to support public decision-making. But ultimately, results depend on scientists' subjective choices (e.g., of statistical models, and interpretations) and can be apprehended only through the prism of social practice.

Emphasizing the need for more theory, Burghardt and Bodansky note that psychology as a science has left the first phase of exploratory research in favor of theory-driven research. To manage this transition, Burghardt and Bodansky present five key challenges. Challenge One is about how to best support researchers to advance the field. Challenge Two concerns psychology's transition from protoscience to paradigmatic science, in which scientists are challenged to develop robust paradigms that help associate and restructure currently unrelated findings and theories. Challenge Three involves a revised methodology needed along the lines of Lakatos, who developed Popper's critical rationalism into a more theory-friendly research advance. Challenge Four stresses the need for harmonizing processes between theory and evidence, in line with Klaus Holzkamp's ideas. Finally, Burghardt and Bodansky present as Challenge Five a 10-point checklist for good research.

Does psychological science have not only a neglected relationship to theory but also a peculiar relationship to methods that are worthy of scrutiny? Several authors agree. For example, Mayrhofer and Hutmacher report on Gerd Jüttemann's so-called “principle of inversion,” which has befallen psychology as a science, by allowing its content to be dictated by methodology. This means a contrast between strict methodological requirements and the comprehensive and often unclear thematic content of psychological research. In other sciences, by contrast, the content is in the foreground. As a consequence, psychological science must abandon the notion of a hierarchy of power in methods, with just quantitative ones at the top. Instead, postmodern science argues for focusing on psychological phenomena that can be understood only through the application of a plurality of methods.

Borgstede and Stolz discuss the different importance of replication in deductive, variable-based, more quantitatively oriented research (top-down generalization) vs. in inductive, case-based, more qualitatively oriented research (bottom-up generalization). If replication fails in the former, the theory would have to be falsified because the approach assumes the need for the generalizability of theories. In less formalized inductive research, in contrast, a failed replication leads to considerations about the limitations of a theory's validity. Borgstede and Stolz argue for a more frequent and open-minded use of bottom-up generalization because statistical sample-based generalization modeling is—from the perspective of formal logic—unattainable in social and psychological science.

Edelsbrunner discusses various statistical and conceptual rationales for generating sum scores across items (e.g., in psychological tests), arguing that any given score can only represent the particular theoretical model that has been used to create it. Therefore, he demands that researchers explain why and how they want to justify the use of scores either through a specific theoretical rationale (e.g., conceptualization or definition) or a specific statistical rationale (e.g., through a statistical model). The general aim of Edelsbrunner is to get beyond long-standing consensus views, such as that traditional latent variable models could be adequate representations underlying any measurement process.

Zitzmann and Loreth critically discuss how researchers' preferences of statistical methods often influence their mutual approval as researchers (e.g., as “being” Frequentist vs. Bayesian) and how this hampers knowledge dissemination (e.g., through overly critical reviews)—and thus scientific progress. They argue for strengthening researchers' shared identity as psychologists (e.g., by facilitating non-mainstream publications in the same respected journals) without having to give up their disapproval of lower levels of identity (e.g., preferences for particular methods). In particular, mutual tolerance and respect of others as equals enable much-needed critical discussion and serious debate.

With an even stronger focus on methodological research practices, Uher analyzes in-depth the philosophical-theoretical foundations on which rating scales are built, revealing a dense network of 12 complexes of problematic concepts, misconceived assumptions, and fallacies. Uher demonstrates how—through the popularity of rating scales and their uncritical interpretation as enabling psychological “measurement”—these problems have become institutionalized in a wide range of psychological practices, thereby perpetuating psychology's crises (e.g., replication, validity, generalizability, and confidence). To tackle these problems holistically, Uher derives from all 12 problem complexes specific theoretical concepts, methodologies, and methods as well as key directions of development, highlighting the necessity to explore individuals as complex living beings and to consider the study phenomena's peculiarities (e.g., momentariness, contextuality, and intra-individual variation) as well as the inherent anthropogenicity of any research on individuals.

Hanfstingl points out that methodology has to develop a new understanding of objectivity to meet future requirements of validity in psychological science. Taking up the argument from feministic and sociological research that different perspectives must be considered to enhance objectivity, Hanfstingl holds that different theories and methods are to be defined as concrete perspectives on a psychological phenomenon. Psychological research methodology has many methodical tools available to systematically apply different perspectives on a phenomenon, such as, for example, specification analyses, meta-analyses, combinatorial meta-analyses, or approaches combining any of those. Hanfstingl argues that research programs should be designed on the base of these formally explicated perspectives and around a psychological phenomenon of interest.

All of these contributions highlight the need for a paradigm shift in psychological science. This shift is thereby not seen as a complete break from what has been done so far but rather as a gradual change developed and implemented over generations of researchers. A pivotal common thread is a need for a greater focus on the integration of new developments in theory, methodology, and methods in order to meet the requirements of future research and contemporary real-world problems. A key insight is that psychological knowledge is much more complex than the mainstream understanding currently represents. Many psychologists' understanding of validity and objectivity is deeply shaken because they are struggling to find consensus even regarding the meaning of “success” in the replication of empirical studies. Consequently, there is a substantial need for new epistemological and methodological developments, in particular, because these developments have not yet reached mainstream research and its ongoing debates. The old ways of doing research often do not work anymore, whereas new developments are not yet fully elaborated and functional.

All the authors of our Research Topic are working toward solutions and they have contributed ideas and strategies for dealing with these new insights that can no longer be ignored in psychological science. These insights call for a closer look at what “modern” science in psychology already offers, at the new directions that “post-modern” and other epistemologies have opened up, and at how other disciplines are already dealing successfully with this change. Many authors also note that psychology has evolved from a protoscience to a paradigmatic science with all the consequences that this entails, at both the theoretical and the methodological level. They call for intensified research on theory in psychology and much greater use of theoretical knowledge to gain new insights. Many authors of our Research Topic ascertained that focusing on the psychological phenomena in themselves rather than just on psychological constructs about them is more helpful in gaining new insights. They offer new variants and alternatives for generating scientific data and interpreting results that meet the complexity of psychological study phenomena more appropriately and that enable the generation of psychological findings with higher validity, replicability, generalizability, and confidence.

To outline such a paradigm shift, we believe that today's understanding of science has changed in several ways compared to the understanding of science by earlier generations of scientists. In Table 1, we summarize the main differences between the traditional “modern” view of psychological science and the recent “postmodern” and further developments of psychological science including some controversial ideas discussed in the articles of our Research Topic without any claim of completeness.

**Table 1:** Comparison of key assumptions of “modern” vs. “postmodern” and further epistemologies in psychology discussed in this Research Topic.

**“Modern” psychology**	**“Postmodern” psychology and beyond**	**Discussed by**
Protoscience	Paradigmatic science	Burghardt and Bodansky
Orientation toward traditional natural sciences	The necessary focus on psychological phenomena and their peculiarities, requiring the involvement of sciences beyond the traditional natural sciences	Burghardt and Bodansky; Guyon; Iso-Ahola; Mayrhofer and Hutmacher; Mayrhofer et al.; Mazur; Rabeyron; Uher
Knowledge generation through authority and scientific hierarchy, focusing on important scientist personalities (“VIPs of science”)	Collective knowledge generation with less focus on influential individual researchers but instead on diversity in researchers and their sociocultural and research backgrounds	Burghardt and Bodansky; Guyon; Hanfstingl; Mayrhofer et al.; Mazur; Uher; Veraksa et al.; Zitzmann and Loreth; conversely discussed by Holtz
Generalized theories are regarded as valid across contexts and populations	Theories and approaches that are valid only locally or temporarily, with this limited validity being regarded as a strength rather than a weakness; Accepting the contextuality of findings	Borgstede and Scholz; Guyon; Hanfstingl; Holtz; Mayrhofer and Hutmacher; Mayrhofer et al.; Iso-Ahola; Mazur; Uher; Veraksa et al.
Knowledge from single studies	Knowledge from meta-analyses, meta-syntheses, and reviews	Hanfstingl; Rabeyron; Uher
Primary focus on empirical studies	Focus on both theory development and empirical studies	Borgstede and Scholz; Burghardt and Bodansky; Edelsbrunner; Guyon; Hanfstingl; Iso-Ahola; Mayrhofer and Hutmacher; Mayrhofer et al.; Mazur; Uher; Veraksa et al.; Zitzmann and Loreth
Implicit hierarchy of the quality of scientific methods with quantitative methods at the top	Plausibility of a method's applicability and its appropriateness to the peculiarities of the study phenomena	Borgstede and Scholz; Guyon; Holtz; Krueger; Mayrhofer and Hutmacher; Mayrhofer et al.; Mazur; Uher; Rabeyron; Zitzmann and Loreth
Focus on only one method, one approach, or one theory	Manifold and complementary use of different methods, approaches, theories or even disciplines to gain new insights	Borgstede and Scholz; Hanfstingl; Holtz; Krueger; Mayrhofer and Hutmacher; Iso-Ahola; Uher; Veraksa et al.; Zitzmann and Loreth
Orientation toward psychological constructs	Orientation toward psychological phenomena in themselves away from beliefs about them as reflected in everyday constructs	Borgstede and Scholz; Burghardt and Bodansky; Edelsbrunner; Guyon; Hanfstingl; Iso-Ahola; Mayrhofer and Hutmacher; Mayrhofer et al.; Mazur; Uher; Veraksa et al.
Replicated studies produce valid knowledge, unreplicated studies produce invalid knowledge	Re-interpretation of replication as a method for examining generalizability and contextuality	Borgstede and Scholz; Iso-Ahola; Mayrhofer et al.; Mazur; Rabeyron; Uher
Statistics as a truth-generator	Re-interpretation of statistics as socio-constructivist activity legitimately dependent on the researcher	Borgstede and Scholz; Burghardt and Bodansky; Edelsbrunner; Guyon; Hanfstingl; Holtz; Iso-Ahola; Mayrhofer and Hutmacher; Mazur; Uher; Zitzmann and Loreth
Rules on how to use methodological principles	Arbitrariness in the use of methodological principles	Holtz; Krueger; Zitzmann and Loreth; conversely discussed by Mayrhofer et al.; Uher
Accepting knowledge as valid because it has been published in peer-reviewed journals	A critical look at the processes of knowledge generation and their transparency	Guyon; Rabeyron

It is our hope that this compilation of research papers will contribute new ideas, theories, concepts, and methodologies to current debates on psychological research practices and will provide good food for thought to help psychologist tackle their current challenges and advance their discipline and its research meaningfully.

## Author contributions

BH and JU developed the idea for this article, drafted, and finalized the manuscript. PE, UD, and TG provided feedback and suggestions. All authors approved the manuscript for submission.

